# TIMPs of parasitic helminths – a large-scale analysis of high-throughput sequence datasets

**DOI:** 10.1186/1756-3305-6-156

**Published:** 2013-05-30

**Authors:** Cinzia Cantacessi, Andreas Hofmann, Darren Pickering, Severine Navarro, Makedonka Mitreva, Alex Loukas

**Affiliations:** 1 Center for Biodiscovery and Molecular Development of Therapeutics, Queensland Tropical Health Alliance, James Cook University, Cairns, Queensland, Australia; 2 Structural Chemistry Program, Eskitis Institute, Griffith University, Brisbane, Queensland, Australia; 3 The Genome Institute, Washington University School of Medicine, St. Louis, MO, USA

**Keywords:** Tissue inhibitors of metalloproteases, Parasitic helminths, Excretory/secretory products, Transcriptomics, Bioinformatics, Protein structure, Functional inferences

## Abstract

**Background:**

Tissue inhibitors of metalloproteases (TIMPs) are a multifunctional family of proteins that orchestrate extracellular matrix turnover, tissue remodelling and other cellular processes. In parasitic helminths, such as hookworms, TIMPs have been proposed to play key roles in the host-parasite interplay, including invasion of and establishment in the vertebrate animal hosts. Currently, knowledge of helminth TIMPs is limited to a small number of studies on canine hookworms, whereas no information is available on the occurrence of TIMPs in other parasitic helminths causing neglected diseases.

**Methods:**

In the present study, we conducted a large-scale investigation of TIMP proteins of a range of neglected human parasites including the hookworm *Necator americanus*, the roundworm *Ascaris suum*, the liver flukes *Clonorchis sinensis* and *Opisthorchis viverrini*, as well as the schistosome blood flukes. This entailed mining available transcriptomic and/or genomic sequence datasets for the presence of homologues of known TIMPs, predicting secondary structures of defined protein sequences, systematic phylogenetic analyses and assessment of differential expression of genes encoding putative TIMPs in the developmental stages of *A. suum, N. americanus* and *Schistosoma haematobium* which infect the mammalian hosts.

**Results:**

A total of 15 protein sequences with high homology to known eukaryotic TIMPs were predicted from the complement of sequence data available for parasitic helminths and subjected to in-depth bioinformatic analyses.

**Conclusions:**

Supported by the availability of gene manipulation technologies such as RNA interference and/or transgenesis, this work provides a basis for future functional explorations of helminth TIMPs and, in particular, of their role/s in fundamental biological pathways linked to long-term establishment in the vertebrate hosts, with a view towards the development of novel approaches for the control of neglected helminthiases.

## Background

Parasitic helminths cause devastating diseases in humans and animals worldwide [1,2]. Amongst these parasites, soil-transmitted helminths (STHs), including *Ancylostoma duodenale* and *Necator americanus* (hookworms), *Ascaris* sp. (roundworms) and *Trichuris* spp. (whipworms), are estimated to infect over one-sixth of all humans [1,2], while trematodes, including the blood flukes *Schistosoma* spp. and the carcinogenic liver flukes *Clonorchis sinensis* and *Opisthorchis viverrini*, affect >200 million people worldwide [3-5].

Traditionally, the control of helminth infections has relied on the repeated and frequent use of anthelmintics [6,7], which is likely to lead to the development of drug resistance against the compounds administered (cf. [8-10]). Indeed, some studies [11-15] have reported a reduction in efficacy of mebendazole and pyrantel in *N. americanus* and *A. duodenale* in areas of Mali, North-Western Australia and Zanzibar, which has been attributed to emerging anthelmintic resistance. Given the limited knowledge of the molecular mechanisms linked to the development of drug resistance in parasitic helminths [16], as well as the unavailability of effective vaccines, much attention is now directed towards the identification of novel targets for intervention [7,17]. A detailed understanding of the molecular biology of parasitic helminths, and in particular of the structure and function of key genes and gene products playing essential roles in host-parasite interactions, could provide a basis for the design of novel therapeutics.

Among several groups of helminth molecules involved in the host-parasite interplay, protease inhibitors have been the subject of intense investigations due to their roles in a range of fundamental molecular processes, including regulation of host proteases and modulation of the host’s immune response [18]. Amongst these molecules, the inhibitors of cysteine- and serine-proteases (= cystatins and serpins, respectively; MEROPS family I25 and I04, respectively) are known to participate in the cascades of molecular events leading to parasite development through the larval stages (cystatins) [19], as well as in the inhibition of host molecules responsible for the initiation of blood coagulation (serpins) [20,21]. In addition, both molecular groups have been proposed to play key roles in the evasion and modulation of the immune response of the vertebrate host [19,21,22]. In contrast to data on cystatins and serpins, knowledge of the biological roles of parasite-derived tissue inhibitors of metalloproteases (TIMPs) is limited. Eukaryote TIMPs are a multifunctional family of inhibitors of matrix metalloproteases (MMPs), including collagenases and gelatinases, which function as important regulators of extracellular matrix (ECM) turnover, tissue remodelling and cellular behaviour [23]. The N-terminal domain of TIMPs contains a netrin module (=‘NTR’; Prosite: PDOC50189) which, in addition to harbouring the functional site responsible for the primary metalloprotease inhibitory activity, is associated with a range of biological roles, including axon guidance, regulation of cell-cell interactions during embryogenesis, cell proliferation, angiogenesis and pro- and anti-apoptotic pathways [23,24]. The NTR domain of TIMPs is also found in other groups of proteins, such as the frizzled-related (PDOC50038) and the laminyn-type EGF-like (PDOC00961) proteins, in which it fulfills distinct biological roles (cf. [24]).

In the canine hookworm *Ancylostoma caninum,* TIMPs are abundant components of the excretory/secretory (ES) products of the adult worm [25-27]. *Ac*-TMP-1 and *Ac*-TMP-2, two abundantly expressed TIMPs recognized by sera from dogs vaccinated with irradiated third-stage larvae (L3s) of *A. caninum*, were proposed to play key roles in the host-parasite interplay [25,27]. To date, no data on the occurrence of TIMP homologues/orthologues in other parasitic helminths is available. Over the last decade, advances in next-generation sequencing (NGS) technologies and bioinformatics [28-31] have provided the infrastructure for large-scale analyses of the genomes and transcriptomes of a range of parasitic helminths of public health significance, including the nematodes *N. americanus*, *Ascaris suum*, *Trichuris suis* and *Trichinella spiralis* (gastrointestinal nematodes) [32-36] and the trematodes *Schistosoma mansoni*, *S. japonicum*, *S. haematobium* (blood flukes), *C. sinensis* and *O. viverrini* (liver flukes) [37-41]. These advances have resulted in an expansion of sequence data available in public databases (e.g., http://www.gasserlab.org/, http://www.genedb.org/, http://www.ncbi.nlm.nih.gov/, http://nematode.net/NN3_frontpage.cgi and http://www.sanger.ac.uk/research/projects/parasitegenomics/), which represent an invaluable resource for studies of TIMPs in parasitic helminths. In the present study, we (i) conducted the first large-scale investigation of TIMP proteins in a range of parasitic helminths of both human and veterinary health significance; (ii) inferred phylogenetic relationships between/among helminth TIMPs based on predictions of secondary structures of protein sequences; and (iii) investigated differences in the levels of transcription of genes encoding putative TIMPs in different developmental stages of *A. suum* (cf. [34]), *N. americanus* (cf. [36]) and *S. haematobium* (cf. [40]).

## Methods

### Sequence data, and identification and bioinformatic analyses of TIMPs

The sequence data obtained from public sequence databases (i.e. National Center for Biotechnology Information at http://www.ncbi.nlm.nih.gov/; ENSEMBL Genome Browser at http://www.ensembl.org/index.html; WormBase, at http://www.wormbase.org; GeneDB at http://www.genedb.org/; http://www.gasserlab.org) [32-34,39,40,42-45] and analysed herein included known TIMP amino acid sequences from *Homo sapiens* (GenBank accession numbers XP_010392.1, NP_003246.1, P35625.1 and Q99727.1), *Mus musculus* (accession numbers P12032.2, P25785.2, P39876.1 and Q9JHB3.1), *Canis familiaris* (AF112115.1), *Gallus gallus* (AAB69168.1), *Oryctolagus cuniculus* (AAB35920.1), *Drosophila melanogaster* (AAL39356.1), *A. caninum* (AF372651.1 and EU523698.1), *A. duodenale* (ABP88131.1) and *Caenorhabditis elegans* (NP_505113.1), as well as predicted peptides inferred from (i) the whole or draft genome sequences of *S. mansoni*, *S. japonicum*, *S. haematobium* (http://www.genedb.org), *A. suum* (http://www.wormbase.org), *T. spiralis* (http://www.ncbi.nlm.nih.gov/nuccore/316979833), *Brugia malayi* and *Wuchereria bancrofti* (human filarial nematodes) (http://www.sanger.ac.uk/; [46]), *N. americanus* (human hookworm; [36]), and (ii) the transcriptomes of *T. suis* (swine whipworm), *Oesophagostomum dentatum* (swine nodule worm) (http://www.gasserlab.org), *Dictyocaulus filaria* (sheep lungworm; [47]) and *C. sinensis*, *O. viverrini* (human liver flukes), *Fasciola hepatica* and *F. gigantica* (bovine and deer liver fluke, respectively) (http://www.gasserlab.org). The algorithms BLASTp [48] and InterProScan [49] were used to identify TIMP proteins in each of the genomic and transcriptomic datasets based on sequence homology (e-value cut-off: 10^-5^) with known TIMP proteins from eukaryotes [50]. In addition, the software pScan (http://www.psc.edu/general/software/packages/emboss/appgroups/pscan.html) was used to identify regular-expression based diagnostic patterns for TIMPs (Prosite: PS00288). Signal peptides were also predicted using the program SignalP 3.0, employing both the neural network and hidden Markov Models [51]. Putative ES TIMP proteins were identified based on the presence of a signal peptide and sequence homology to one or more known ES proteins listed in the Secreted Protein (http://spd.cbi.pku.edu.cn/; [52]) and the Signal Peptide (http://proline.bic.nus.edu.sg/spdb/index.html; [53]) databases.

### Secondary structure predictions and homology modelling

Structure-based sequence alignments of TIMP proteins were computed and manually edited with SBAL [54] guided by secondary structure elements predicted using the PSIPRED software [55]. Individual structure-based alignments of amino acid sequences were subjected to analysis by Bayesian inference (BI) using the program MrBayes v.3.1.2 [56] and verified by Maximum Likelihood analysis using the program MEGA v.5 [57] and the Jones-Taylor-Thornton substitution model with uniform rates among sites (JTT + G + I). Each BI analysis was conducted for 1,000,000 generations (ngen = 1,000,000), with every 100-th tree being saved, using the following parameters: rates = gamma, aamodelpr = mixed, and the other parameters left at the default settings. Tree and branch lengths were measured employing the parameter ‘sumt burnin = 1000’; an unrooted, consensus tree was constructed, with ‘contype = halfcompat’ nodal support being determined using consensus posterior probabilities and displayed employing the software FigTree (http://tree.bio.ed.ac.uk/software/figtree/). For selected TIMPs, homologues with known three-dimensional structures were identified using the protein-fold recognition software pGenTHREADER [58] and selected as templates for comparative modelling using MODELLER [59]. Twenty independent models were generated, and the model with the lowest energy was selected, its geometry analysed using PROCHECK [60] and then inspected visually with PyMOL [61].

### Assessment of levels of transcription of TIMP-encoding genes

The raw sequence reads derived from each of the non-normalized cDNA libraries from *A. suum* infective L3s (iL3s; from eggs), migrating L3s (from liver and lung), fourth-stage larvae (L4s, from the small intestine) and muscular and reproductive tissues from each adult male and female [34], *N. americanus* iL3s and adults (mixed males and females) [36], as well as *S. haematobium* eggs and adult male and female [40] were mapped to the longest contigs encoding individual putative TIMP proteins using the program SOAP2 [62]. Briefly, raw sequence reads were aligned to the non-redundant transcriptomic data, such that each raw sequence read was uniquely mapped (i.e. to a unique transcript). Reads that mapped to more than one transcript (designated ‘multi-reads’) were randomly assigned to a unique transcript, such that they were recorded only once. To provide a relative assessment of transcript abundance, the number of raw reads that mapped to each sequence was normalized for length (i.e. reads per kilobase per million reads, RPKM) [34,40,63].

## Results and discussion

### TIMP proteins of parasitic helminths

A total number of 15 protein sequences with high homology (e-value cut-off: 10^-5^) to known eukaryotic TIMPs were predicted from the complement of sequence data available for parasitic helminths (Table 1), thus representing a solid resource for future structural and functional investigations of this protein family in parasites. The sequence data in FASTA format analysed in the present article is available in Additional file 1. Of the datasets included here, the complement of protein coding genes available for *N. americanus* and *A. suum* encoded the largest number of predicted TIMP proteins (*n* = 8 and 3, respectively; cf. Table 1). Three *N. americanus* (i.e. NECAME_13168, NECAME_07191 and NECAME_08458; cf. Table 1) and all *A. suum* TIMPs (GS_21732, GS_04796 and GS_08199; cf. Table 1) were predicted to contain an N-terminal signal peptide, in accordance with previous observations of *A. caninum Ac*-TMP-1 and *Ac*-TMP-2 and a netrin-domain containing homologue from *Ancylostoma ceylanicum* (= excretory-secretory protein 2, AceES-2), respectively [25-27,64]. Despite the sequence similarities between *Ac*-TMP-1, *Ac*-TMP-2 and AceES-2, the latter did not display human MMP inhibitory activity *in vitro*, thus suggesting a different function of this protein *in vivo*[64]. However, it should be noted that the partial MMP-inhibitory activity of *Ac*-TMP-2 described by Zhan *et al.*[26] was based on a vast molar excess of recombinant TMP-2, well beyond the 1:1 inhibitor:enzyme molar ratio required for inhibition of mammalian MMPs by their TIMP counterparts [23]. Moreover, TIMPs seem to require the C-X-C motif at the N-terminus to allow insertion into the MMP active site cleft and subsequent inhibition of catalytic activity; recombinant *Ac*-TMP-2 was engineered to contain a long N-terminal extension donated by the plasmid vector, so it is premature to unequivocally assign MMP inhibitory activity to the hookworm TIMPs without further work. In *A. ceylanicum*, secretion of AceES-2 begins soon after infection of the experimental hamster host, and steadily increases in correspondence with the onset of blood-feeding activity [65]. Furthermore, a single oral dose of recombinant AceES-2 resulted in reduced anaemia following challenge infection of hamsters with *A. ceylanicum*[66], which led to speculations that this molecule may play a role in the pathogenesis of hookworm disease [66]. A role for hookworm TIMPs in molecular processes linked to the invasion of the mammalian hosts and/or the inhibition of hosts MMPs at the final site of attachment has also been hypothesized, based on the fact that *Ac*-TMP-2 could be isolated solely from extracts and ES products of *A. caninum* adults, despite the corresponding mRNA being detected from both L3s and adults of this parasite [26].

**Table 1:** Number of tissue inhibitor of metalloproteases (TIMP) and netrin module (NTR)-containing protein sequences, respectively, identified in each sequence dataset and listed according to taxa

	**TIMPs (no. with SP)**	**NTR-module containing proteins (no. with SP)**
Nematodes		
*Ascaris suum*	3* (3)	2 (1)
*Brugia malayi*	-	1 (1)
*Dictyocaulus filaria*	1 (1)	1 (−)
*Necator americanus*	8** (3)	1 (1)
*Oesophagostomum dentatum*	2 (2)	1 (−)
*Trichinella spiralis*	-	1 (1)
*Trichuris suis*	-	2 (−)
*Wuchereria bancrofti*	-	2 (−)
Trematodes		
*Clonorchis sinensis*	-	1 (−)
*Fasciola gigantica*	-	2 (−)
*Fasciola hepatica*	-	1 (−)
*Opisthorchis viverrini*	-	4 (1)
*Schistosoma haematobium*	1*** (1)	5 (−)
*Schistosoma japonicum*	-	1 (−)
*Schistosoma mansoni*	-	1 (−)
Total	15 (10)	26 (5)

The number of proteins containing a predicted N-terminal signal peptide (SP) is also indicated.

*Of these, *Ascaris suum* GS_04796 was up-regulated in the reproductive tissue of the adult female, while GS_21732 was up-regulated in the muscular tissue of adult males.

**Necator americanus NECAME_13168 and NECAME_07191 were up-regulated in infective L3s, while NECAME_08457 and NECAME_08458 were up-regulated in adult *N. americanus,* ***Schistosoma haematobium A_01727 was up-regulated in the adult male.

Of the eight genes encoding putative TIMPs in *N. americanus*, transcription of NECAME_13168 and NECAME_07191 was significantly up-regulated in iL3s (cf. Table 1; [36]), thus supporting a role for these proteins in the infection process of the human host. Conversely, NECAME_08457 and NECAME_08458 displayed high transcription levels in adult *N. americanus* (cf. Table 1; [36]), which likely reflects a diversification of function of members of this protein family in different developmental stages of this parasite. In the future, studies of differential transcription of genes encoding TIMPs in both genders and different tissues of *N. americanus* may help elucidate the roles that these molecules play in the fundamental molecular biology of the adult nematode. In *A. suum*, transcription of GS_04796 was significantly up-regulated in the adult female reproductive tissue of this nematode, whereas GS_21732 was up-regulated in the male muscle (cf. Table 1; cf. [34]). The putative TIMP proteins encoded by GS_04796 and GS_21732 share ~40% similarity with *C. elegans* CRI-2 (WBGene00019478; http://www.wormbase.org), the expression of which has been localized to the body wall musculature and to the vulval, anal and pharyngeal muscles of the adult nematode (cf. http://www.wormbase.org). In *C. elegans*, *cri-2* is known to function in the cascade of molecular events linked to the regulation of the innate immune response to lipopolysaccharide (LPS) [67]. In a previous study, inhibition by small interfering RNAs (siRNAs) of the *M. musculus* ortholog of *C. elegans cri-2* in a mouse macrophage cell line stimulated with *Escherichia coli* LPS resulted in decreased production of interleukin-6 (IL-6) [67]. This cytokine, *in vivo*, is associated with a wide range of biological activities, which include the generation of acute-phase reactions in response to infections by pathogens [68]. The putative role/s that parasite homologs of *C. elegans cri-2* play in the modulation of innate immunity in vertebrate hosts remain/s unknown. However, recent evidence that recombinant *Ac*-TMP-1 promotes the development of a regulatory immune response by modifying the functions of bone marrow-derived dendritic cells and subsequent development of regulatory T cells [69], supports a key role for this TIMP in establishing an anti-inflammatory environment.

In flatworms, the *S. haematobium* gene A_01727 encoded the only trematode TIMP protein that could be identified using computational methods. Analysis of transcriptional regulation of *S. haematobium* A_01727 in different developmental stages revealed that this molecule is up-regulated in the adult male of this parasitic trematode (Table 1; cf. [40]). The transcript encoding mouse TIMP-1 is up-regulated in male gonads during testis morphogenesis, while expression of the corresponding protein was restricted to the cords of foetal testes [70]. In addition, the human and mouse genes encoding TIMP-2 are known to include the differential display clone 8 (DDC8) gene, whose transcription is enhanced during spermatogenesis [71]. These observations, together with earlier findings of increased expression of TIMP-1 in human foetal Sertoli cells [72,73] and testicular expression of TIMP-2 in rats [74], led to the hypothesis that these molecules may play specific roles during testis organogenesis and development [70], as well as in the migration of germ cells through the seminiferous epithelium [71]. Therefore, it is tempting to speculate a role for *S. haematobium* A_01727 in biological processes linked to the reproductive activity of the adult male fluke; however, this hypothesis requires rigorous testing. In the future, genetic manipulation of *N. americanus*, *A. suum* and *S. haematobium* by RNA interference (RNAi) and/or transgenesis [75-78], may help elucidate the function/s of putative helminth TIMPs in the reproductive biology of these organisms, as well as in other fundamental molecular processes, for instance those linked to host invasion and modulation of the host’s innate immune response.

**Figure 1:**
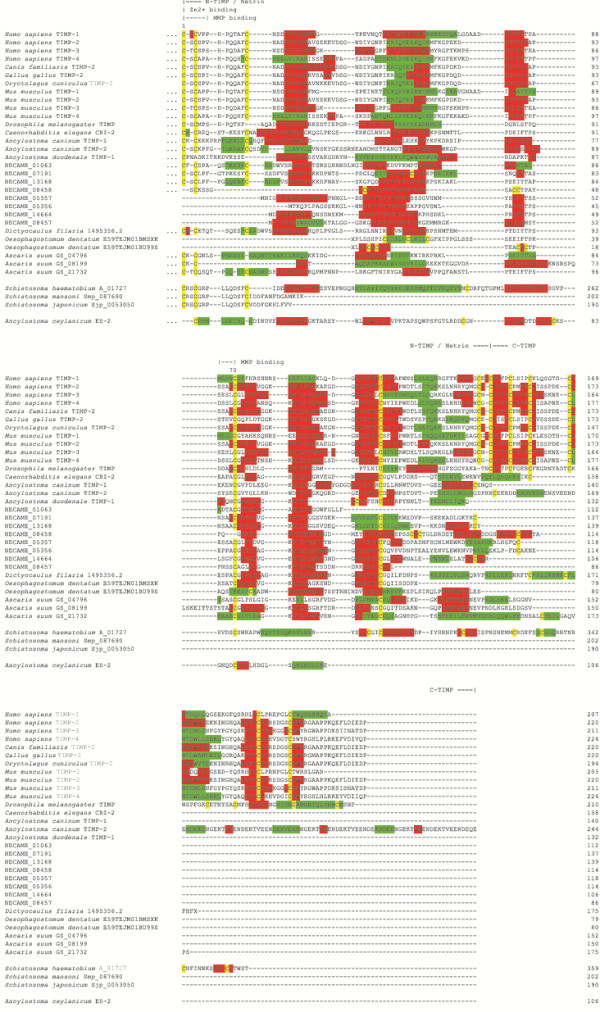
**Amino acid sequence alignment of tissue inhibitors of metalloproteases (TIMPs) based on predictions of their secondary structures.***Homo sapiens* TIMP-1 (GenBank accession number XP_010392.1), TIMP-2 (NP_003246.1), TIMP-3 (P35625.2), TIMP-4 (Q99727.1), *Canis familiaris* TIMP-2 (AF112115.1), *Gallus gallus* TIMP-2 (AAB69168.1), *Oryctolagus cuniculus* TIMP-2 (AAB35920.1), *Mus musculus* TIMP-1 (P12032.2), TIMP-2 (P25785.2), TIMP-3 (P39876.1), TIMP-4 (Q9JHB3.1), *Drosophila melanogaster* TIMP (AAL39356.1), *Caenorhabditis elegans* CRI-2 (K07C11.5), *Ancylostoma caninum* TMP-1 (AF372651.1), TMP-2 (EU523696.1), *Ancylostoma duodenale* TIMP-1 (ABP88131.1), *Necator americanus* (NECAME_13168, NECAME_07191, NECAME_01063, NECAME_05356, NECAME_05357, NECAME_14664, NECAME_08457 and NECAME_08458), *Dictyocaulus filaria* (1495356.2; http://www.gasserlab.org), *Oesophagostomum dentatum* (E59TEJM01BU99S and E59TEJM02GRTKW; http://www.gasserlab.org), *Ascaris suum* (GS_21732, GS_04796, GS_08199; http://www.wormbase.org), *Schistosoma haematobium* A_01727, *Schistosoma mansoni* Smp_087690 and *Schistosoma japonicum* Sjp_0053050 (http://www.genedb.org). *Ancylostoma ceylanicum* AceES-2 (GenBank Q6R7N7) is also included.

Genomic sequence data with identity to *S. haematobium* A_01727 were detected in both *S. mansoni* (Smp_087690; e-value 3e-110) and *S. japonicum* (Sjp_0053050.1; e-value 6.3e-64). However, the sequence overlap between the amino acid sequence predicted from *S. haematobium* A_01727 and the corresponding homologues from *S. mansoni* and *S. japonicum* was limited to the NTR N-terminal module (cf. Figure 1), which would make any inference of the presence of TIMP-encoding genes in the genome sequences of the latter two species highly speculative. While it is possible that fragmentation of the Open Reading Frames (ORFs) of TIMP-encoding genes in the current assemblies of the *S. mansoni* and *S. japonicum* genomes might have occurred, the absence of homologues of eukaryote TIMPs in other species whose whole-genome sequences are currently available (e.g. *B. malayi* and *T. spiralis*) may reflect the substantial variations, both in sequence and in length, among members of this protein family in helminths [23]. Indeed, a search of the characteristic features of the N-terminal NTR module of eukaryote TIMPs using the PScan software revealed the presence of members of the netrin protein family in all parasitic helminths analysed herein (*n* = 26; range 1–5; cf. Table 1). This finding is in accordance with current knowledge that the genomes of helminths encode single-domain TIMP proteins that are homologous to the N-terminal domain of vertebrate TIMPs, while lacking the corresponding C-terminal region [79]. In eukaryotes, the N-terminal NTR domain of TIMPs is known to be responsible for their metalloprotease inhibitory activity [24,80,81], whereas the C-terminal domain provides binding sites for the metalloproteases [80,82,83] or for binding TIMPs to the cell surface and/or the extracellular matrix [24,81,84]. When separated from the corresponding C-terminus, the N-terminal domain of TIMPs retains its metalloprotease inhibitory activity [24,81-84]. While, based on this knowledge, single-domain helminth TIMPs may be hypothesized to exert similar metalloprotease inhibitory activities as their vertebrate counterparts, the amino acid residues present at position 2 of some mature helminth molecules (e.g. lysine, arginine and glutamine; cf. Figure 1) are atypical for vertebrate TIMPs and suggest that these proteins may perform functions that are unrelated to the inhibition of metalloprotease activity (see [23,85]). Comparative structural analyses of the amino acid sequences of TIMP proteins, as well as the N-terminal NTR module are essential to assist in-depth investigations of the function/s of this family of helminth proteins.

### Structural analyses of eukaryote TIMPs

Structurally, the four human TIMPs are well characterized (cf. http://www.rcsb.org). These proteins consist of two domains, an N-terminal domain (N-TIMP) adopting the NTR fold, and a C-terminal domain (C-TIMP). Tertiary structures of full-length TIMP-1, TIMP-2, as well as N-TIMP-1, N-TIMP-2 and N-TIMP-3 have been determined, some in complex with their target MMPs (for an overview, see Table 2). Both N-TIMP and C-TIMP are internally stabilised by three intra-domain disulphide bridges and their structural elements are not intertwined, suggesting that the two moieties are indeed individual folding units, i.e. domains. This notion is further supported by the observation that N-TIMPs can be obtained as folded entities *in vitro* that display MMP inhibitory activity [79,86-88]. The shape of full-length TIMPs appears wedge-like, and the extreme N-terminus is responsible for the inhibitory action of MMPs by interaction with the protease active site cleft. In some instances, additional interactions have been observed between C-TIMP and peripheral areas of the protease that are distant to the catalytic site. However, in the case of the TIMP-2/MMP-2 complex, the interaction of C-TIMP-2 and the hemopexin domain of MMP-2 significantly enhances the affinity of the inhibitor [89,90].

**Table 2:** Three-dimensional structures of tissue inhibitors of metalloproteases (TIMPs) and their complexes available in the Protein Data Bank (PDB; http://www.rcsb.org/pdb/home/home.do) as of Nov 2012

**Protein(s)**	**PDB accession code**
N-TIMP-1	1d2b
MMP1:TIMP-1	2j0t
MMP3:TIMP-1	1uea
MMP3:N-TIMP-1	1oo9
MMP10:TIMP-1	3v96
MMP14:TIMP-1	3ma2
TIMP-2	1br9
N-TIMP-2	2tmp
pro-MMP2:TIMP-2	1gxd
MMP13:TIMP-2	2e2d
MMP14:TIMP-2	1bqq
MMP14:TIMP-2	1buv
TACE:N-TIMP-3	3cki

The main interactions of TIMPs with their target proteases are formed by a continuous peptide at the N-terminal end (Cys1-Pro5 in human TIMP-1) and in a loop connecting two adjacent β-strands (Met66-Cys70 in human TIMP-1). The two regions are covalently linked by a disulphide bond (Cys1-Cys70 in human TIMP-1), and are located in the netrin module (N-TIMP) of the protein which adopts the fold of a five-stranded α-barrel with Greek key topology (OB-fold) flanked by two α-helices. The N-terminus of N-TIMP inserts into the active site of the target protease and the α-amino and the carbonyl group of Cys-1 (human TIMP-1) coordinate the active site zinc ion of the protease by displacing a water molecule otherwise bound to the metal [23]. Residue 2 (Ser, Thr) projects into the specificity (S1′) pocket of the protease. Residues 3–5 interact with the protease residues in the primed subsites, which normally harbour substrate residues C-terminal of the scissile bond. Similarly, residues 66–70 of TIMP-1 occupy the non-primed subsites of the protease that otherwise interact with the residues N-terminal to the scissile bond.

As apparent from the structure-based amino acid sequence alignment (Figure 1), TIMPs from parasitic helminths are characterised by higher sequence variation than their mammalian homologues, in accordance with the results of previous analyses of invertebrate TIMPs [23]. With respect to structure-function relationships, however, the most important feature grafted onto the netrin fold seems to be the conformation neighbouring Cys-1. In vertebrate TIMPs, 2 is either a serine or threonine that projects into the protease specificity pocket. It is important to note that neither *Ac*-TMP-1 nor *Ac*-TMP-2 have been convincingly shown (via 1:1 inhibitor:enzyme molar ratios) to possess MMP inhibitory activity. Moreover, AceES-2 produced with a flush N-terminus was screened for MMP activity at 15:1 and 115:1 molar ratios and did not display inhibitory activity (cf. [64]). The amino acid sequence alignment in Figure 1 highlights the general motif of TIMPs, C-X-C, in this region. It shows for the helminth TIMP with published inhibitory activity, *Ac*-TMP-2, that in addition to serine and threonine, lysine is a tolerated residue at position 2 for inhibition. Notably, AceES-2 and *Ad*-TIMP-1 from *A. duodenale* lack the second cysteine residue as well as a suitable residue at position 2 (Ser/Thr/Lys) able to protrude into the S1′ pocket of the protease for inhibition (cf. Figure 1). On this basis, one would predict *Ad*-TIMP-1 to not have any MMP-inhibitory activity. Thus, helminth TIMPs that show conservation at position 2 are likely to display inhibitory activities against human MMPs. The *S. haematobium* protein encoded by A_01727 possesses two residues (Arg-Ser) between the two N-terminal cysteine residues, which makes the prediction of functional effects difficult in the absence of experimental structures.

**Figure 2:**
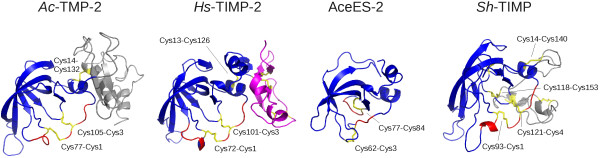
**Structural comparison of four netrin domain-containing proteins.** The netrin domains of *Ac*-TMP-2 (homology model based on *Hs*-TIMP-2), *Hs*-TIMP-2 (PDB accession code 1br9), AceES-2 (PDB accession code 3nsw) and *Sh*-TIMP (A_01727; homology model based on *Hs*-TIMP-2) are coloured blue, cysteine side chain residues are rendered as yellow sticks. Red highlighted areas indicate regions of interactions with MMPs; these regions are inferred for *Ac*-TMP-2, AceES-2 and *Sh*-TIMP based on the alignment in Figure 1. The parasite proteins *Ac*-TMP-2 and *Sh*-TIMP and human *Hs*-TIMP-2 share the same intra-domain disulphide bonding pattern. In contrast, AceES-2 possesses a different pattern with two intra-molecular disulphide bonds. The disulphide bond engaging the N-terminal cysteine (Cys3-Cys62) is reminiscent of that found in *Ac*-TMP-2, *Sh*-TIMP and *Hs*-TIMP-2. The other disulphide bond (Cys77-Cys84) is unique to AceES-2. The C-terminal domain of *Hs*-TIMP-2 is rendered magenta. The C-terminal domains of *Ac*-TMP-2 and *Sh*-TIMP are shown in grey for illustration only and the three-dimensional structures of these domains are neither based on computational nor experimental evidence. Comparative modelling was performed using MODELLER [59] based on the structure-based sequence alignment shown in Figure 1.

Helminth TIMPs for which complete amino acid sequence data is available, with the exception of *Ad*-TIMP-1, show conservation of the crucial structural elements of the NTR module, such as the two N-terminal cysteine residues and their covalent binding partners, as well as residues relevant for maintaining the OB-fold. The areas of largest variation are three surface-exposed loop areas, namely residues 28–41, 56–59 and 66–70 (*Hs*-TIMP-2 numbering; see Figure 1). Notably, there is high conservation of a basic residue (Arg20 in *Hs-*TIMP-1) in vertebrate and helminth TIMPs, which is an exposed residue on the surface distal to the protease interaction site (Figure 2). To our knowledge, a physiologically important function for this residue is yet to be described. Its location (at the surface of the protein) suggests a protein-protein or protein-matrix interaction; however, basic residues at this position have not been reported to be involved in extra-cellular matrix binding [91]. While *S. haematobium* A_01727 shares the lowest amino acid sequence identity with the other eukaryote TIMPs (cf. Figure 1), the structure-based sequence alignment, together with the accordingly predicted 3D structure, indicate that it may be a functional member of the TIMP family of proteins. This conclusion is based on the presence of all conserved cysteine residues required for intramolecular disulphide bonds of a netrin-like fold, as well as conservation of the serine residue (Ser3) expected to protrude into the catalytic site of an MMP.

### Phylogenetic analysis

**Figure 3:**
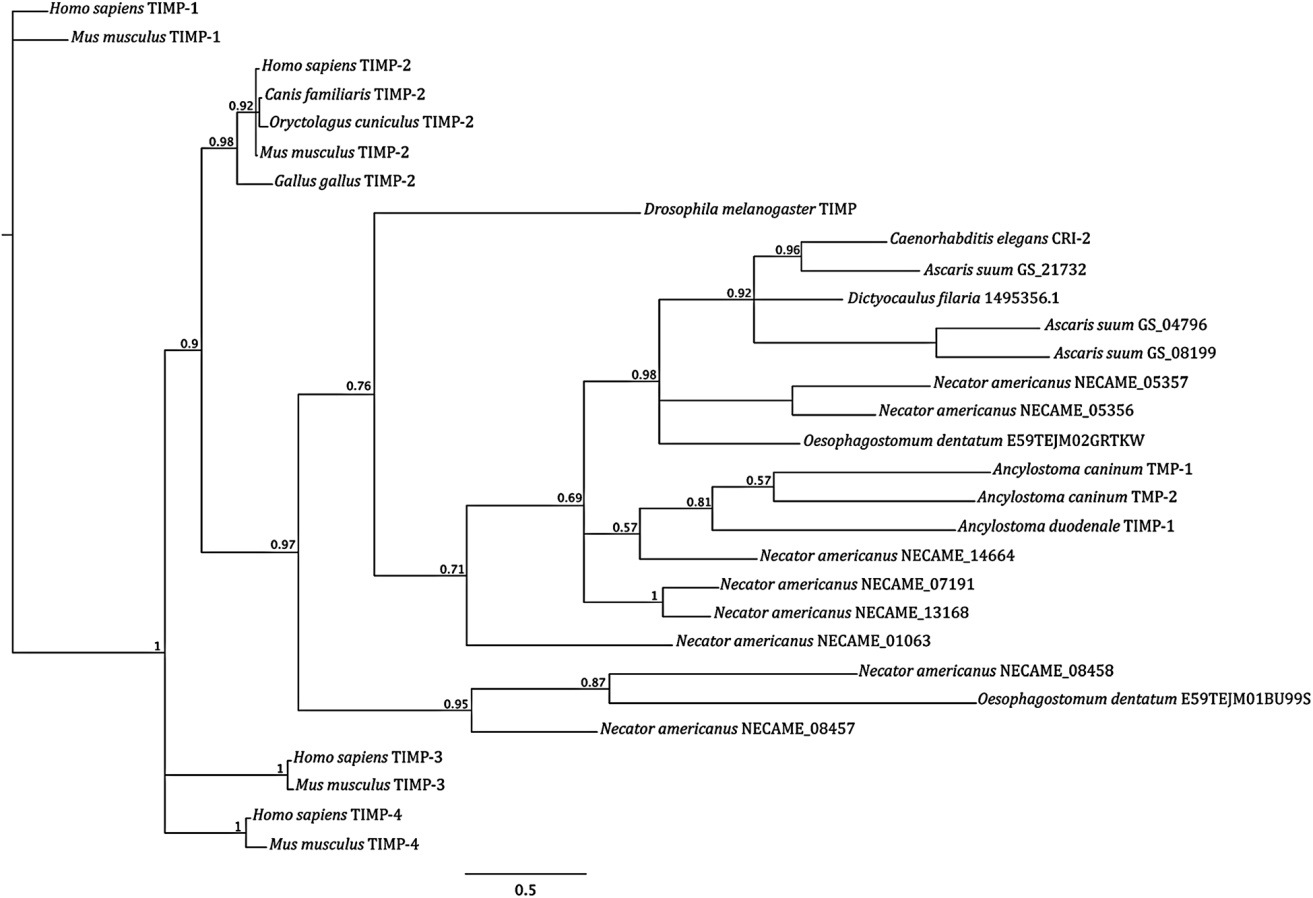
**The phylogenetic relationships of tissue inhibitor of metalloproteases (TIMPs) based on Bayesian Inference.** The posterior probability supporting each clade is indicated. The corresponding phylogenetic reconstructions obtained by Maximum Likelihood (JTT + G + I) analyses of TIMP proteins are available from the primary author (CC) upon request. *Homo sapiens* TIMP-1 (GenBank accession number XP_010392.1), TIMP-2 (NP_003246.1), TIMP-3 (P35625.2), TIMP-4 (Q99727.1), *Gallus gallus* TIMP-2 (AAB69168.1), *Canis familiaris* TIMP-2 (AF112115.1), *Oryctolagus cuniculus* TIMP-2 (AAB35920.1), *Drosophila melanogaster* TIMP (AAL39356.1), *Mus musculus* TIMP-1 (P12032.2), TIMP-2 (P25785.2), TIMP-3 (P39876.1), TIMP-4 (Q9JHB3.1), *Caenorhabditis elegans* CRI-2 (K07C11.5), *Ancylostoma caninum* TMP-1 (AF372651.1), TMP-2 (EU523696.1), *Ancylostoma duodenale* TIMP-1 (ABP88131.1), *Necator americanus* (NECAME_13168, NECAME_07191, NECAME_01063, NECAME_05356, NECAME_05357, NECAME_14664, NECAME_08457 and NECAME_08458), *Dictyocaulus filaria* (1495356.2; http://www.gasserlab.org), *Oesophagostomum dentatum* (E59TEJM01BU99S and E59TEJM02GRTKW; http://www.gasserlab.org) and *Ascaris suum* (GS_21732, GS_04796, GS_08199; http://www.wormbase.org).

The phylogenetic analysis of eukaryote TIMPs allowed us to study the relationships between helminth TIMPs and their vertebrate counterparts (Figure 3). The analysis identified one main clade comprising TIMPs from invertebrates, including free-living and parasitic helminths (nodal support: 0.90), to the exclusion of clades formed by homologues from vertebrates (cf. Figure 3). Within the invertebrate clade, a sub-clade representing TIMPs from nematodes clustered to the exclusion of the TIMP protein from *D. melanogaster* (nodal support: 0.76; cf. Figure 3), supporting the existence of a monophyletic group of TIMPs for parasitic nematodes. Following the inclusion of *S. haematobium* A_01727 in the phylogenetic analysis, the monophyly of the nematode TIMP clade with respect to the vertebrate homologues was maintained (Additional file 2). No distinct separation between TIMPs from hookworms and those from other free-living and parasitic nematodes was observed, thus supporting the hypothesis that nematode TIMPs may be characterised by specific functional properties, distinct from those of their vertebrate homologues. Whether nematode TIMPs have originated following loss of the C-terminal domain from a vertebrate ancestor or from a distinct gene line (cf. [23]) remains to be explored.

## Conclusion

The current availability of ‘-omics’ technologies, applied to in-depth investigations of pathogens causing neglected diseases [31,92-94], are becoming pivotal for a better understanding of the structure and function of TIMP proteins in different species and developmental stages of parasitic helminths. For instance, data from in-depth comparative structural analyses between helminth TIMPs and their vertebrate counterparts, will be crucial in future studies aimed at assessing the suitability of parasite TIMPs as novel targets for intervention. Supported by the availability of the whole-genome sequences of, for instance, schistosomes and *A. suum*[34,37,38,40] and by current efforts to expand genomic sequencing to other neglected parasites (e.g. hookworms; [95]), the application of gene manipulation technologies such as RNAi and/or transgenesis [94,96,97], will allow the function of helminth TIMP proteins in fundamental biological pathways to be elucidated. Perhaps the most important question that is yet to be addressed in any depth is the function of helminth TIMPs. Are they inhibitors of metalloproteases? Is their primary purpose to suppress inflammation, and if so, how do they do it? We hope that the molecular information provided herein on parasitic helminth TIMPs will provide a framework on which to build intensive research activities around this intriguing family of proteins and their roles in host-parasite interactions.

## Competing interests

The authors declare that they have no competing interests.

## Authors’ contributions

CC, AH and AL conceived and designed the experiments; CC, AH, DP and SN analysed the data; CC, AH, MM and AL wrote the manuscript. All authors read and approved the final version of the manuscript.

## Supplementary Material

Additional file 1 TIMP amino acid sequences inferred from genomic and/or transcriptomic sequence data analysed in the present study. Click here for file

Additional file 2 **Phylogenetic relationships.** The phylogenetic relationships of tissue inhibitor of metalloproteases (TIMPs) based on Bayesian Inference. The posterior probability supporting each clade is indicated. The corresponding phylogenetic reconstructions obtained by Maximum Likelihood (JTT + G + I) analyses of TIMP proteins are available from the primary author (CC) upon request. *Homo sapiens* TIMP-1 (GenBank accession number XP_010392.1), TIMP-2 (NP_003246.1), TIMP-3 (P35625.2), TIMP-4 (Q99727.1), *Gallus gallus* TIMP-2 (AAB69168.1), *Canis familiaris* TIMP-2 (AF112115.1), *Oryctolagus cuniculus* TIMP-2 (AAB35920.1), *Drosophila melanogaster* TIMP (AAL39356.1), *Mus musculus* TIMP-1 (P12032.2), TIMP-2 (P25785.2), TIMP-3 (P39876.1), TIMP-4 (Q9JHB3.1), *Caenorhabditis elegans* CRI-2 (K07C11.5), *Ancylostoma caninum* TMP-1 (AF372651.1), TMP-2 (EU523696.1), *Ancylostoma duodenale* TIMP-1 (ABP88131.1), *Necator americanus* (NECAME_13168, NECAME_07191, NECAME_01063, NECAME_05356, NECAME_05357, NECAME_14664, NECAME_08457 and NECAME_08458Contig150, Contig565, Contig7; http://www.gasserlab.org), *Dictyocaulus filaria* (1495356.2; http://www.gasserlab.org), *Oesophagostomum dentatum* (E59TEJM01BU99S and E59TEJM02GRTKW; http://www.gasserlab.org) *Ascaris suum* (GS_21732, GS_04796, GS_08199; http://www.wormbase.org) and *Schistosoma haematobium* (A_01727; http://www.genedb.org). Click here for file
