# Supplementary figures and images for: TIMPs of parasitic helminths – a large-scale analysis of high-throughput sequence datasets
Source: Parasit Vectors. 2013 May 30;6:156. doi: 10.1186/1756-3305-6-156 (PMC3679795; doi:10.1186/1756-3305-6-156)

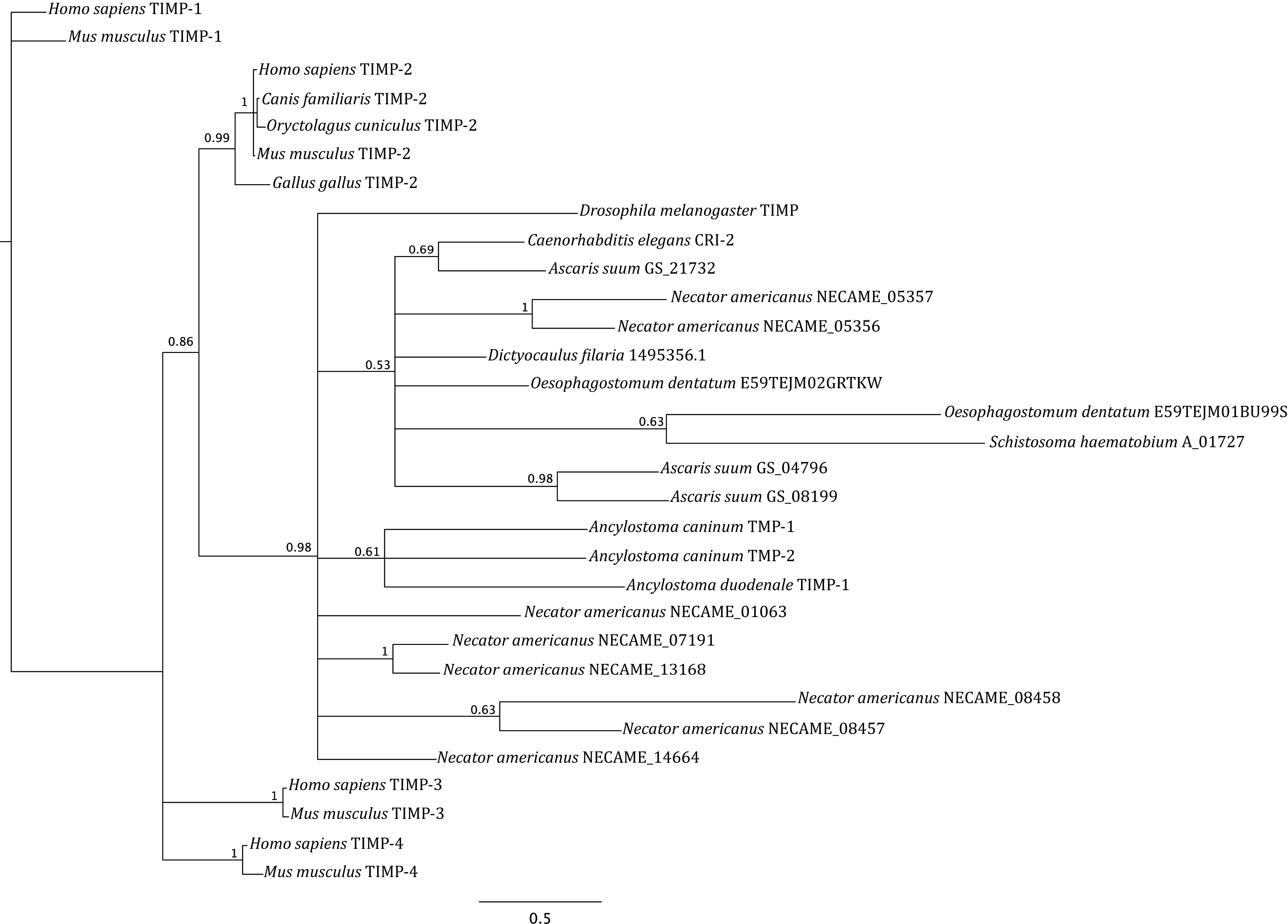

Supplement: Additional file 2 — Phylogenetic relationships. The phylogenetic relationships of tissue inhibitor of metalloproteases (TIMPs) based on Bayesian Inference. The posterior probability supporting each clade is indicated. The corresponding phylogenetic reconstructions obtained by Maximum Likelihood (JTT + G + I) analyses of TIMP proteins are available from the primary author (CC) upon request. Homo sapiens TIMP-1 (GenBank accession number XP_010392.1), TIMP-2 (NP_003246.1), TIMP-3 (P35625.2), TIMP-4 (Q99727.1), Gallus gallus TIMP-2 (AAB69168.1), Canis familiaris TIMP-2 (AF112115.1), Oryctolagus cuniculus TIMP-2 (AAB35920.1), Drosophila melanogaster TIMP (AAL39356.1), Mus musculus TIMP-1 (P12032.2), TIMP-2 (P25785.2), TIMP-3 (P39876.1), TIMP-4 (Q9JHB3.1), Caenorhabditis elegans CRI-2 (K07C11.5), Ancylostoma caninum TMP-1 (AF372651.1), TMP-2 (EU523696.1), Ancylostoma duodenale TIMP-1 (ABP88131.1), Necator americanus (NECAME_13168, NECAME_07191, NECAME_01063, NECAME_05356, NECAME_05357, NECAME_14664, NECAME_08457 and NECAME_08458Contig150, Contig565, Contig7; http://www.gasserlab.org), Dictyocaulus filaria (1495356.2; http://www.gasserlab.org), Oesophagostomum dentatum (E59TEJM01BU99S and E59TEJM02GRTKW; http://www.gasserlab.org) Ascaris suum (GS_21732, GS_04796, GS_08199; http://www.wormbase.org) and Schistosoma haematobium (A_01727; http://www.genedb.org). [file 1756-3305-6-156-S2.tiff]
